# Analysis of kinetoplast cytochrome *b* gene of 16 *Leishmania* isolates from different foci of China: different species of *Leishmania* in China and their phylogenetic inference

**DOI:** 10.1186/1756-3305-6-32

**Published:** 2013-02-05

**Authors:** Bin-Bin Yang, Da-Li Chen, Jian-Ping Chen, Lin Liao, Xiao-Su Hu, Jia-Nan Xu

**Affiliations:** 1 Department of Medical Laboratory, Weifang Medical University, #7166, The West Baotong street, Weifang, Shandong, 261053, China; 2 Department of Parasitology, West China School of Preclinical and Forensic Medicine, Sichuan University, #17, The 3th Section of South Renmin Road, Chengdu, Sichuan, 610041, China; 3 Animal Disease Prevention and Food Safety Key Laboratory of Sichuan Province, #24, The South 1st Section of Yihuan Road, Chengdu, Sichuan, 610041, China

**Keywords:** *Leishmania*, Phylogeny, cyt *b*, China, *Sauroleishmania*

## Abstract

**Background:**

*Leishmania* species belong to the family Trypanosomatidae and cause leishmaniasis, a geographically widespread disease that infects humans and other vertebrates. This disease remains endemic in China. Due to the large geographic area and complex ecological environment, the taxonomic position and phylogenetic relationship of Chinese *Leishmania* isolates remain uncertain. A recent internal transcribed spacer 1 and cytochrome oxidase II phylogeny of Chinese *Leishmania* isolates has challenged some aspects of their traditional taxonomy as well as cladistics hypotheses of their phylogeny. The current study was designed to provide further disease background and sequence analysis.

**Methods:**

We systematically analyzed 50 cytochrome *b* (cyt *b*) gene sequences of 19 isolates (16 from China, 3 from other countries) sequenced after polymerase chain reaction (PCR) using a special primer for cyt *b* as well as 31 sequences downloaded from GenBank. After alignment, the data were analyzed using the maximum parsimony, Bayesian and netwok methods.

**Results:**

Sequences of six haplotypes representing 10 Chinese isolates formed a monophyletic group and clustered with *Leishmania tarentolae*. The isolates GS1, GS7, XJ771 of this study from China clustered with other isolates of *Leishmania donovani* complex. The isolate JS1 was a sister to *Leishmania tropica,* which represented an *L. tropica* complex instead of clustering with *L. donovani* complex or with the other 10 Chinese isolates. The isolates KXG-2 and GS-GER20 formed a monophyletic group with *Leishmania turanica* from central Asia. In the different phylogenetic trees, all of the Chinese isolates occurred in at least four groups regardless of geographic distribution.

**Conclusions:**

The undescribed *Leishmania* species of China, which are clearly causative agents of canine leishmaniasis and human visceral leishmaniasis and are related to *Sauroleishmania*, may have evolved from a common ancestral parasite that came from the Americas and may have split off earlier than the other old world *Leishmania*. Our results also suggest the following: the isolates GS7, GS1 and XJ771 occur as part of the *L. donovani* complex; the JS1 isolate is *L. tropica;* and the isolate GS-GER20 identified as *Leishmania gerbilli* is close to KXG-2 which is *L. turanica*.

## Background

The leishmaniases are a group of vector borne diseases that are caused by flagellate of the genus *Leishmania,* which is transmitted by the bite of the sandfly, and affect as many as 12 million people worldwide with 1.5–2 million new cases each year in 88 countries [1]. The genus *Leishmania* consists of nearly 30 species of morphologically similar kinetoplastid protozoa, and approximately 20 of these species are responsible for a spectrum of human diseases that ranges from mild to fatal infections [2,3].

It is well accepted now that the genus *Leishmania* forms a monophyletic group with three distinct subgenera *Leishmania* (*Leishmania*), *Leishmania* (*Viannia*), and *Leishmania* (*Sauroleishmania*) [4]. Once, the *Leishmania*-like parasites of reptiles were recognized as a separate genus *L.* (*Sauroleishmania*) [5,6]. But the classification of lizard *Leishmania* as subgenus *L*. (*Sauroleishmania*) was proposed by Saf’janova [7], Croan *et al.*[8], Orlando *et al.*[9], Zelazny *et al.*[10] and Fraga *et al.*[4] on the basis of biological criteria and analysis of different *Leishmania* gene. According to the absolute chromosomal size difference index (aCSDI) analysis of as many as 31 “conserved” chromosomes, subgenera *L*. (*Leishmania*) is divided into clusters: (1) Old world representatives of subgenus *Leishmania* (OWL), and (2) New world representatives of subgenus *Leishmania* (NWL) and subgenus *Viannia* (NWV) [6].

The complexity of the taxonomy and phylogenetic relationships of the Chinese *Leishmania* was due to the extensive geographic area and complex ecological environment. Identification of species responsible for different leishmaniasis and clinical manifestation remains uncertain. The strains from cutaneous leishmaniasis (CL) in Xinjiang Uygur Autonomous Region (Xinjiang) especially in Karamay is closely related to *L. tropica* with analysis of SSU rDNA gene[11,12], whereas the pathogen identified as *Leishmania infantum*[13,14] or *Leishmania turanica*[15] from the same geographic region Karamay also could cause CL. However, *L. turanica* is nonpathogenic to humans, according to Strelkova *et al.*[16]. The parasites of some visceral leishmaniasis (VL) cases in Sichuan and Gansu provinces were *L. donovani* or undescribed species *Leishmania* sp. [17-19]. VL and CL have been reported in China to date the species of *Leishmania* comprises much more than that. The isolates in China were more heterogeneous than previously thought, requiring the reassignment of some isolates into different groups as described by Lu *et al.*[20].

Over the past few decades, DNA markers including coding and non-coding genes have become additional information for advancing our understanding of evolutionary and phylogenetic relationships and species differentiation. Data pertaining to the ribosomal RNA (rRNA) gene sequences–in particular, the two non-coding, highly variable internal transcribed spacer regions (ITS1 and ITS2) are considered to be acceptable molecular criteria for resolving taxonomic questions and determining the phylogenetic affinities among closely related *Leishmania* species [17,21-25]. While coding genes are also wildly used for taxonomic studies, such as metabolic enzymes (ICD, ME, MPI, G6PDH, ASAT, GPI, NH1, NH2, PGD and FH) [26,27], heat-shock protein 70 gene (hsp70) [4], cytochrome oxidase II (CO II) [28], the gene encoding the largest subunit of RNA polymerase II (rpoIILS) [6,8], the glycoprotein 63 gene (*gp*63) [29], cysteine protease B genes (*cpb*) [30] and cytochrome *b* (cyt *b*) [31-35].

In previous ITS1 and CO II study [17,19], we summarized the four endemic *Leishmania* species in China: *L. donovani*, *L. infantum*, *Leishmania gerbilli*, and *L. turanica*. We also noted that there might be an undescribed *Leishmania* species endemic in China and highlighted that the isolate IPHL/CN/77/XJ771 from Bachu County, Xinjiang, is *L. donovani* instead of *L. infantum*. To elucidate the phylogeny, evolution and epidemiology of interesting group of strains in China, further studies of more genes are required.

Cyt *b* is one of the cytochromes involved in the electron transport process of the mitochondrial respiratory chain is considered one of the most useful genes for phylogenetic work [34]. Marco *et al.* proved that the cyt *b* gene sequencing can precisely identify the *Leishmania* spp. for all of the local stocks that are well characterized by multi-locus enzyme electrophoresis (MLEE), the current gold standard [32]. Phylogeny and sequence variation of the genus *Leishmania* has also been discussed successfully with cyt *b* sequencing [34,35]. In this paper, the cyt *b* gene of *Leishmania* from China was sequenced and analyzed using bioinformatics methods. Moreover, the phylogenetic relationships were reconstructed using cyt *b* sequences obtained by this study and download from the GenBank database. We then discuss in detail the implications of relationships between strains in China and other locations.

## Methods

### *Leishmania* isolates

A population of cloned promastigotes (including 16 Chinese *Leishmania* isolates and three isolates from other countries) was stored in liquid nitrogen, and kept at the Department of Parasitology, Western China School of Preclinical and Forensic Medicine, Sichuan University. All of the *Leishmania* isolates used in this study are listed in Table 1. The promastigotes were cultivated in medium 199 supplemented with 15% heat-inactivated fetal bovine serum (HIFBS) at 28°C. Approximately 1–5 × 10^9^ promastigotes were collected at room temperature by centrifugation at 3300 × g for 10 min and washed with phosphate-buffered saline.

**Table 1:** **List of *Leishmania *strains, origin, and database accession numbers, including sequences of *Leishmania *retrieved from GenBank**

**Sequence length (bp)**	**GenBank accession numbers**	**MLEE-based species assignment**	**WHO code**	**Origin**	**Reference**
1079	HQ908255^a^	*Leishmania* sp.	MHOM/CN/84/SD1	Shandong, China	This study
1078	HQ908260^a^	*Leishmania* sp.	MHOM/CN/90/SC10H2	Sichuan, China	This study
1079	HQ908263^a^	*Leishmania* sp.	MHOM/CN/89/GS6	Gansu, China	This study
1079	HQ908264^a^	*Leishmania* sp.	MHOM/CN/86/SC6	Sichuan, China	This study
1080	HQ908271^a^	*Leishmania* sp.	MHOM/CN/84/GS3	Gansu, China	This study
1080	HQ908273	*Leishmania* sp.	MHOM/CN/83/GS2	Gansu, China	This study
1079	HQ908266	*Leishmania* sp.	MHOM/CN/80/XJ801	Xinjiang, China	This study
1079	HQ908272	*Leishmania* sp.	MHOM/CN/90/SC11	Sichuan, China	This study
979	HQ908268	*Leishmania* sp.	MCAN/CN/86/SC9	Sichuan, China	This study
1079	HQ908269	*Leishmania* sp.	MHOM/CN/89/GS5	Gansu, China	This study
1080	HQ908259	*L. gerbilli*	MGER/CN/60/GS-GER20	Gansu, China	This study
1080	HQ908256	*L. turanica*	MRHO/CN/88/KXG-2	Karamay, China	This study
1079	HQ908262	*L. donovani* complex	MCAN/CN/60/GS1	Gansu, China	This study
1079	HQ908261	*L. donovani* complex	MHOM/CN/93/GS7	Gansu, China	This study
1079	HQ908267	*L. donovani* complex	IPHL/CN/77/XJ771	Xinjiang, China	This study
1060	HQ908265	*L. tropica*	MHOM/CN/84/JS1	China	This study
1080	HQ908270^b^	*L. tropica*	MHOM/SU/74/K27	Soviet Union	This study
1080	HQ908257^b^	*L. tropica*	-	-	This study
1080	AB095960^b^	*L. tropica*	MHOM/SU/58/Strain OD	Soviet Union	Luyo-Acero *et al.*, 2004 [35]
1079	AB095965^c^	*L. garnhami*	MHOM/VE/76/JAP78	Venezuela	Luyo-Acero *et al.*, 2004 [35]
1078	HQ908258^c^	*L. mexicana*	-	-	This study
1079	AB095957^d^	*L. donovani*	MHOM/SD/62/2S-25M-C2	Sudan	Luyo-Acero *et al.*, 2004 [35]
1079	AB095958^d^	*L. infantum*	MHOM/TN/80/IPT1	Tunisia	Luyo-Acero *et al.*, 2004 [35]
1080	AB095959^e^	*L. chagasi*	MHOM/BR/74/PP75	Brazil	Luyo-Acero *et al.*, 2004 [35]
872	EF579896^e^	*L. donovani*	MHOM/IN/80/DD8	India	Foulet *et al.*, 2007 [33]
1079	AB434677	*L. archibaldi*	MHOM/ET/72/GEBRE1	Ethiopia	Asato *et al.*, 2009 [34]
872	EF579897^f^	*L. chagasi*	MHOM/BR/74/PP75a	Brazil	Foulet *et al.*, 2007 [33]
872	EF579913^f^	*L. infantum*	MCAN/GR/94/CRE69	Greece	Foulet *et al.*, 2007 [33]
1080	AB095962	*L. aethiopica*	MHOM/ET/72/L100	Ethiopia	Luyo-Acero *et al.*, 2004 [35]
872	EF579908	*L. aethiopica*	MHOM/ET/72/L100	Ethiopia	Foulet *et al.*, 2007 [33]
1080	AB095970	*L. major*	MHOM/EC/88/PT-115	Ecuador	Luyo-Acero *et al.*, 2004 [35]
1080	AB434675	*L. turanica*	MRHO/SU/80/CLONE3720	Soviet Union	Asato *et al.*, 2009 [34]
1080	AB434674	*L. arabica*	MPSA/SA/83/JISH220	Saudi Arabia	Asato *et al.*, 2009 [34]
1104	M97357	*L. mexicana*	-	-	Lee *et al.*, 1992 [36]
1089	M92829	*L. mexicana*	-	-	Lee *et al.*, 1992 [36]
1078	AB095964	*L. amazonensis*	MHOM/BR/73/M2269	Brazil	Luyo-Acero *et al.*, 2004 [35]
1079	AB095963^g^	*L. mexicana*	MHYC/BZ/62/M379	Belize	Luyo-Acero *et al.*, 2004 [35]
872	EF579909^g^	*L. amazonensis*	LMAMPRO/BR/72/M1841	Brazil	Foulet *et al.*, 2007 [33]
872	EF579902^g^	*L. amazonensis*	MHOM/BR/73/M2269	Brazil	Foulet *et al.*, 2007 [33]
1078	AB434678	*L. aristidesi*	MORY/PA/69/GML	Panama	Asato *et al.*, 2009 [34]
1078	M10126	*L. tarentolae*	-	-	de la Cruz *et al.*, 1984 [37]
1078	AB095966	*L. braziliensis*	MHOM/BR/75/M2904	Brazil	Luyo-Acero *et al.*, 2004 [35]
1078	AB434682	*L. braziliensis*	MHOM/BR/75/M2903	Brazil	Asato *et al.*, 2009 [34]
1078	AB095967	*L. braziliensis*	MHOM/EC/88/INH-03	Ecuador	Luyo-Acero *et al.*, 2004 [35]
872	EF579905^h^	*L. guyanensis*	MHOM/GF/79/LEM85	French Guiana	Foulet *et al.*, 2007 [33]
872	EF579912^h^	*L. guyanensis*	MHOM/BR/75/M4147	Brazil	Foulet *et al.*, 2007 [33]
1078	AB095968	*L. panamensis*	MHOM/BR/71/LS94	Brazil	Luyo-Acero *et al.*, 2004 [35]
1078	AB434680	*L. shawi*	MHOM/BR/79/M15065	Brazil	Asato *et al.*, 2009 [34]
1080	AB434686	*L. equatorensis*	MCOH/EC/82/LSP-1	Ecuador	Asato *et al.*, 2009 [34]
1078	M94286	*Trypanosoma brucei*	-	-	Feagin *et al.*, 1987 [38]

a, b, c, d, e, f, g, and h shared the same haplotype.

### DNA extraction and polymerase chain reaction (PCR)

Total genomic DNA of the parasite was extracted by proteinase K digestion and phenol/chloroform/isoamyl alcohol extraction procedures followed by ethanol precipitation to purify the extracted DNA as described by Sambrook and Russell [39]. PCR was performed to generate a fragment spanning cyt *b* kinetoplast DNA (kDNA) between the forward primer COIIIF (5^′^-TAATACGACTCACTATAGTTTATATTG ACATTTTGTWGATT-3^′^) and the reverse primer MURF4R (5^′^- GGGTTTTCCCAG TCACGACGAATCTCTCTCTCCCTT −3^′^) [35]. The PCR protocols for amplification were: 94°C for 3 min followed by 35 cycles of 94°C for 30s, 58°C for 30s, and 72°C for 1.5 min, followed by a final elongation step at 72°C for 10 min. The amplified products were purified on a 2.0% agarose gel stained with ethidium bromide, using a commercial DNA purification kit according to the manufacturer’s protocol*.* The purified PCR product was then sequenced. The DNA sequences of each individual and each species were deposited in the GenBank database under accession numbers (HQ908255-HQ908273).

### Sequence alignment and analyses

A set of cyt *b* sequences of *Leishmania* were retrieved from GenBank, included 29 sequences of genus *Leishmania* (AB095957–AB095960, AB095962–AB095968, AB095970, EF579896–EF579897, EF579902, EF579905, EF579908, EF579909, EF579912, EF579913, AB434674, AB434675, AB434677, AB434678, AB434680, AB434682, M97357, M92829, M10126), *Leishmania equatorensis* (AB434686) and *Trypanosoma brucei* (M94286) (Table 1). The sequences were first aligned using Clustal X 1.83 [40] with a gap-opening penalty of 5 and gap-extension penalty of 1 following the small gap costs recommendation of Hickson *et al.*[41]. The aligned matrix from this procedure was verified to have the same length, and minor adjustments were then made manually using SeaView v.4.2.5 [42]. The data matrices are available from the corresponding author. The haplotype analyses were performed to 37 sequences using DAMBE software for 50 sequences [43]. Compositional heterogeneity was evaluated using chi-square (*χ*2) tests implemented in PAUP* 4.0b10 [44]. Distances from the predicted amino acid sequences were determined with the p-distance models which were computed by MEGA v. 4.1 [45].

### Phylogenetic analyses

Phylogenetic hypotheses of *Leishmania* were generated with cyt *b* kDNA segments using two types of commonly applied phylogenetic techniques: heuristic searches using maximum parsimony (MP) analyses performed with the program PAUP* program and Bayesian inference (BI) using the MrBayes v.3.2 program [46]. In both MP and BI analyses, gaps were treated as missing data. For heuristic searches under parsimony, invariant characters were removed from the dataset. Each search involved 10 random additional replicates, one tree held at each step, with tree bisection and reconnectin branch swapping, steepest descent on, and a maximum of 10,000 saved trees. Non-parametric bootstrapping was used to generate phylogeny confidence values [47], with 1,000 pseudoreplicates using a heuristic tree search for each pseudoreplicate. *Trypanosoma brucei* (M94286) was used to root the trees.

Prior to Bayesian analyses, the best-fit model of evolution, TIM3 + G, was selected using jModeltest v. 0.1.1 [48] under the Bayesian information criterion [49], following recent recommendations [50]. We estimated the posterior probability distributions by allowing four incrementally heated Markov chains (default heating values) to proceed to four million generations, and with samples were taken every 200 generations. Analyses were repeated beginning with different starting trees to ensure that the analyses were not restricted from the global optimum [51]. Convergence was first tested by examining the average deviation of the split frequencies of the two runs, in order to determine whether the two runs had converged. MCMC convergence was also explored by examining the potential scale reduction factor (PSRF) convergence diagnostics for all model parameters (provided by the sump and sumt commands). The first one million generations before this chain became stationary were discarded, and the remaining samples from the independent runs were pooled to obtain the final approximation of the posterior tree distribution.

Sequence alignments were additionally inferred from uncorrected p-distances through NJ networks (Neighbor Net) obtained by SplitsTree 4 [52,53]. This software can detect the alternative evolutionary paths supported by the sequence alignments, and as such, they do not enforce the single bifurcating dendrogram. To yield a single phylogeny hypothesis, the posterior distribution was summarized as a 50% majority rule consensus.

## Results

### Base composition and nucleotide substitution patterns

The size of the newly determined cyt *b* fragments is shown in the Table 1. Of the 911 aligned characters, 324 were variable, including 225 that were parsimony-informative. Percentage base compositions were as follows: A, 27.8; C, 7.2; G, 15.6; and T, 50.4. The average maximum likelihood estimated Ti/Tv ratio was 1.2.

A base stationarity test showed insignificant differences among the taxa in base composition bias in the data (*χ*^2^ = 85.386150, df = 108, p = 0.94687017). The p-distances among the 10 isolates (10 isolates, SD1, SC10H2, GS6, SC6, GS3, GS2, XJ801, SC11, SC9, GS5; the isolates SD1, SC10H1, GS6, SC6 and GS3 share the same sequence) in China were ranged from 0.000 to 0.023 (mean = 0.010), which are smaller than the distances between these isolate and any other known species. These 10 isolates were then classified into the *Leishmania* sp. group. The divergence between *Leishmania* sp. and other *Leishmania* species ranged from 0.051 (*Leishmania* sp. versus *L.tarentolae*) to 0.131 (*Leishmania sp. vs. L. turanica* and *L. gerbilli*), with an average of 0.096, a value that is larger than that within *Leishmania* sp. group. The *L. donovani* complex group contains seven haplotypes: GS1, GS7, XJ771, PP75a (the same sequence with CRE69), 2S-25M-C2 (the same sequence shared with IPT1), PP75 (the same sequence with DD8). The distance within the strains of *L. donovani* complex ranged from 0.001 (the strain GS7 of China *vs. L. chagasi* PP75a and *L. infantum* CRE69) to 0.013 (*L. archibaldi* GEBRE1 *vs. L. chagasi* PP75 and *L. donovani* DD8), which are smaller than the distances between these strains and other known species. The average distance in this group as a whole is 0.006. The p distances among all species except *Leishmania* sp. and *T. brucei* were from 0.002 (between *L. turanica* and *L. gerbilli*) to 0.136 (between *Leishmania arabica* and *L. equatoresnsis*). Most pairwise comparisons mentioned above had divergence values < 0.136, with an average of 0.106 (Table 2).

**Table 2:** **Pairwise genetic distances for cyt*b *segments among *Leishmania *species**

		**1**	**2**	**3**	**4**	**5**	**6**	**7**	**8**	**9**	**10**	**11**
1	*Leishmania* sp.	-										
2	*L. tarentolae*	0.051	-									
3	*L. braziliensis* complex	0.095	0.095	-								
4	*L. mexicana* complex	0.104	0.106	0.107	-							
5	*L. donovania* complex	0.110	0.113	0.102	0.095	-						
6	*L.turaniaca*	0.131	0.131	0.122	0.107	0.092	-					
7	*L. gerbilli*	0.131	0.131	0.123	0.107	0.093	0.002	-				
8	*L. arabica*	0.130	0.130	0.124	0.106	0.101	0.085	0.085	-			
9	*L*. *tropica* complex	0.116	0.119	0.113	0.095	0.081	0.088	0.088	0.095	-		
10	*L. equatoresnsis*	0.126	0.128	0.119	0.112	0.120	0.126	0.127	0.136	0.125	-	
11	*Trypanosoma brucei*	0.177	0.176	0.150	0.158	0.158	0.183	0.183	0.185	0.176	0.155	-

Pairwise genetic distances between different groups are detected by MEGA4.

### Phylogenetic relationships

**Figure 1:**
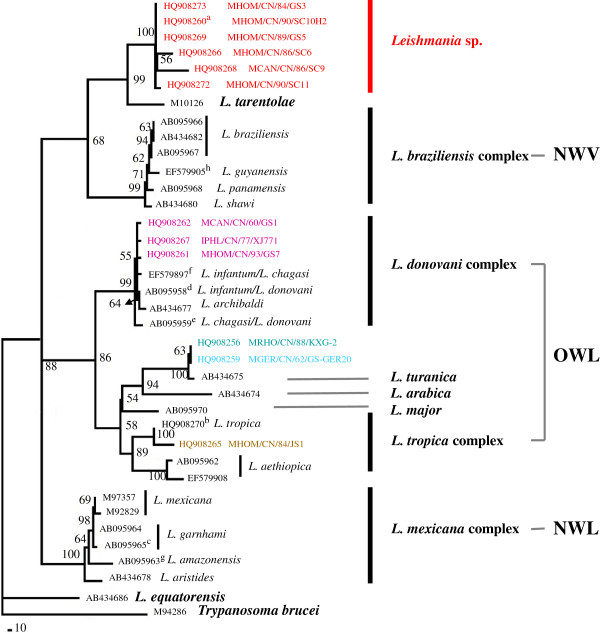
Maximum parsimony consensus tree of the cyt *b *dataset by using PAUP*.*Trypanosoma brucei* (M94286) is the outgroup. Tree length = 15519, CI = 0.6112, RI = 0.8427. The tree is based on haplotypes (identical haplotypes are presented by one strain). The numbers above the branch represent percent recovery in bootstrap analysis (1,000 pseudoreplicates), only bootstrap values >50% are shown. Strains information is shown in Table 1. The letters (a, b, c, d, e, f, g, and h) indicate sharing of a haplotype.

The heuristic search of the cyt *b* matrix resulted in 10,000 equally parsimonious trees of 15519 steps with high CI (0.6112) and RI (0.8427) velues. In the strict consensus phylogram (Figure 1), three clades (BP = 88%) were formed. *Leishmania* sp., *L. tarentolae* and *L. braziliensis* complex form a clade (BP = 68%); six haplotypes in China formed a strongly cluster *Leishmania* sp*.* (BP = 88%), and clustered with with *L. tarentolae* (BP = 99%); and next joined by the *L. braziliensis* complex (BP = 99%) containing three haplotypes of *L. braziliensis*, *L. guyanensis*, *L. panamensis* and *L. shawi*. The OWL clade (BP = 86%) clustered with the following OWL species: *L. donovani*, *L. major*, *L. tropica*, *L. arabica*, *L. turanica* and *L. gerbilli*. *Leishmania chagasi* (with the synonymous *L. infantum*), *L. donovani*, *Leishmania archibaldi* (with the synonymous *L. donovani*) [54], *L. infantum*, and the isolates GS7, GS1 and XJ771of this study from China formed a monophyletic clade *L. dovovani* complex. The strain JS1 from Jiangsu province of China clustered with *L. tropica* from the Soviet Union (BP = 100%) and then clustered with *L. aethiopica* (BP = 100%), which formed the *L. tropica* complex except *L. major* (BP = 89%). *L. turanica* from the Soviet Union clustered with strains GS-GER20 and KXG-2 (BP = 63%) in China from this study clustered together (BP = 100%), which next joined by *L. arabica* (BP = 94%) and *L. major* (BP = 54%). The clade NWL (BP = 100%) is clustered with *L. mexicana*, *L. garnhami*, *L. amazonensis*, and *Leishmania aristides*. Information about the strains is shown in Table 1.

**Figure 2:**
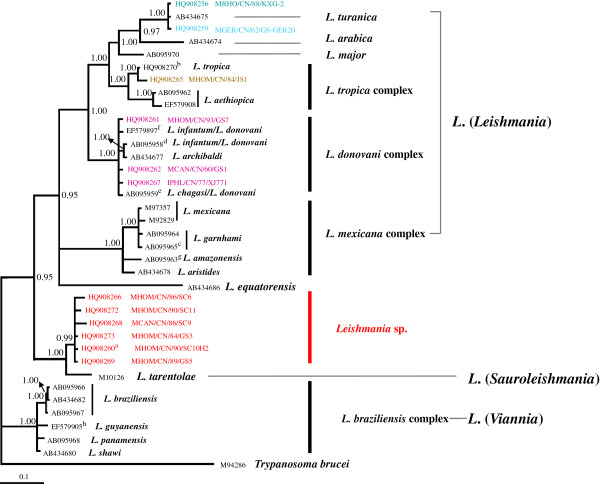
**The 50% majority-rule consensus tree inferred from Bayesian inference of cyt *b *dataset using MrBayes v. 3.2.** The numbers at the nodes represent Bayesian posterior probabilities; TIM3 + G was selected using jModeltest v. 0.1.1. *Trypanosoma brucei* (M94286) is the outgroup. The tree is based on haplotypes (identical haplotypes are presented by one strain). Strain information is shown in Table 1. The letters (a, b, c, d, e, f, g, and h) indicate sharing of a haplotype.

**Figure 3:**
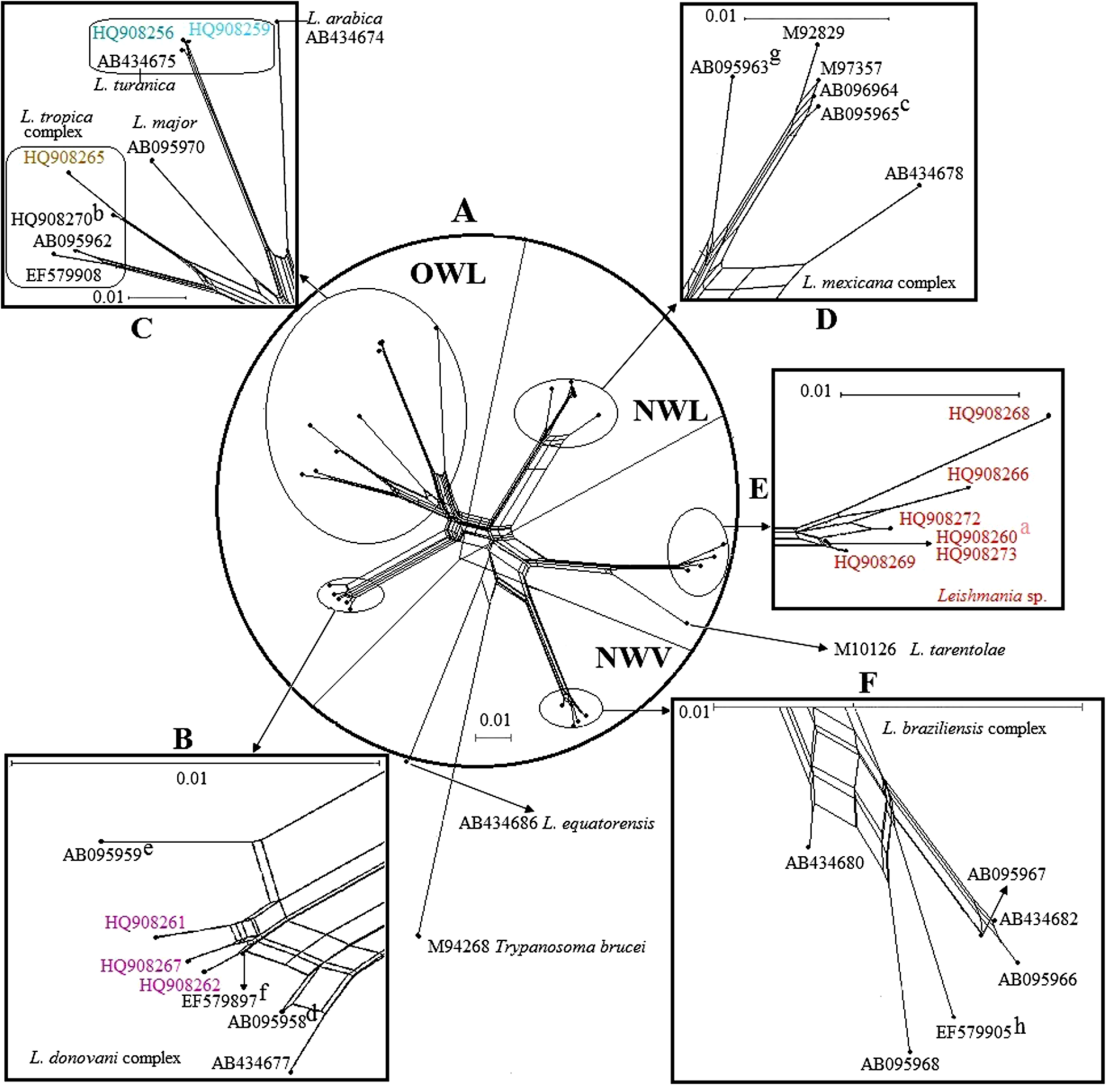
**The phylogenetic network of the *Leishmania *cyt *b *sequences shown in Table **1** and Figures **1**,**2** was built with 1000 bootstrap replicates.** It was algorithm, excluding all conserved site. Distances were calculated using the Kimura 2-parameter distance. *Trypanosoma brucei* (M94286) is the outgroup. Each A-F panel is drawn to the scale indicated and expressed as dissimilarity per nucleotide counted over variable sites (Figures 1–2) in cyt *b* alignment. The dots indicate the sequence position in the network. **A**: Complete network with representation of the five groups shown in detail in the remaining panels; the whole network excluding the *L. equatorensis and T. brucei* is divided into four segments: OWL, NWL, the *Leishmania* sp. and *L. tarentolae* group, and NWV. **B**: The *L. donovani* complex includes three Chinese isolates. **C**: Includes the species *L. tropica*, *L. aethiopica*, *L. major*, *L*. *turanica*, *L. gerbilli*, and *L. arabica.***D**: The New World *Leishmania* subgenus. **E**: The *Leishmania* sp. of this study. **F**: The *L.* (*Viannia*) subgenus. Strain information is shown in Table 1.

For the BI analyses, the likelihood value of the 50% majority consensus tree (Figure 2) was ln L = −4132.1156, while the average PSRF was 1.001. The topology of the BI tree is a little different from that of MP tree. Two robust clades were formed. In one robust clade (PP = 0.95), *Leishmania* sp. (PP = 0.99) was a sister of *L. tarentolae* (PP = 1.00) and forms one clade with the species of *L*. (*Leishmania*) (LL), the other clade was consis of the species of *L*. (*Viannia*) (LV) (PP = 1.00) (see Figure 3). The group of *Leishmania* sp*.* and *L. tarentolae* was basal to subgenus *Leishmania and L. equatorensis*. *L. mexicana*, *L. garnhami*, *L. amazonensis*, and *L. aristidesi* formed the *L. mexicana* complex (PP = 1.00). The *L. mexicana* and *L. equatorensi*s are fundamental to all remaining subgenus *Leishmania* species. Within the other members of subgenus *Leishmania*, the *L. donovani* complex is sister to the clade (PP = 1.00) that clustered with *L. tropica* (PP = 1.00) plus *L. aethiopica* (PP = 1.00), and the clade (PP = 0.97) that consists of *L. turanica, L. gerbilli* and *L. arabica*, next joined by *L. major* (PP = 1.00).

In addition to the common phylogenetic relationships among the different species shown by the MP tree and BI tree, the network (Figure 3) calculated by SplitsTree 4 also indicated a clear evolutionary path with a high value. *Leishmania* sp. and *L. tarentolae* share most of their evolutionary paths.

## Discussion

As a part of worldwide *Leishmania* population, the phylogenetics of Chinese isolates with analysis of the cyt *b* genetic sequences of 16 *Leishmania* isolates was discussed in this paper which demonstrated similarities and differences compared with previous data [17,19] and keep the genus evolutionary unity and integrity over large geographic ranges and time periods.

### *Leishmania* sp. of China

Most interestingly 10 Chinese strains, representing 6 closely related haplotypes, could not be assigned to any of the so far described species of *Leishmania*, a finding that is congruent with our earlier ITS1 and COII studies [17,19]. These *Leishmania* sp. isolates were most closely related to the lizard-infecting *L. tarentolae* (Figures 1, 2, 3).

It was reported that one of these isolates SC6 was collected from patients with VL in Nanping County of Sichuan Province, was infected successfully 8 dogs (8/12) and its amastigotes were detected in their bone marrow smears [55]. Another isolate SC10H2 was proved that it clustered with the pathogen of canine leishmaniasis in Beichuan County, Sichuan Province, China based on the 17S RNA gene [18]. The non-pathogenic to humans *L. tarentolae* has been classified as subgenus *L*. (*Sauroleishmania*) on the basis of biological criteria and different genes [4,8,10,34]. In such cases, we can conclude that the undescribed *Leishmania* species which is clearly a causative agent of canine leishmaniasis and human VL do exist in China are related to the *Sauroleishmania*. However, the more lizard parasites are required to confirm whether *Leishmania* sp. is assigned to the *Sauroleishmania*.

The pairwise genetic distance analysis (Table 2) and phylogenetic network (Figure 3) suggest that the cyt *b* sequences of the Chinese*/tarentolae* group (*Leishmania* sp. and *L. tarentolae*) are closer to the *Viannia* clade than the older world *Leishmania*. This finding is in contrast to that of our ITS1 study [17] and other studies: as an OWL species branching from within New World taxa, *L*. *tarentolae* (*Sauroleishmania*) are closer to the *Leishmania* subgenus than to the *Viannia* subgenus based on different DNA marks (polA and RNA polymerase II, 7SL RNA, hsp 70) [4,8,10]. It is well knows that different genes can have different evolutionary histories and be influenced by selection and horizontal gene transfer, and the phylogenies are also prone to sampling bias; therefore, more genes of diverse geographic original strains would be needed to elucidate the phylogeny, evolution, and epidemiology of the Chinese*/*tarentolae group.

**Figure 4:**
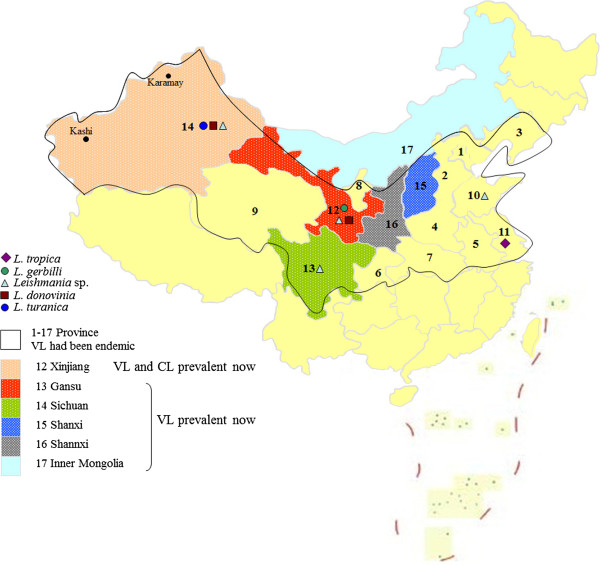
**Distribution of leishmaniasis in China and the specimen collection areas.** 1: Beijiang and Tianjin; 2: Hebei; 3: Liaoning; 4: Henan; 5: Anhui; 6: Chongqing; 7: Hubei; 8: Ningxia Hui Autonomous Region; 9: Qinghai; 10: Shandong; 11: Jiangshu; 12: Gansu; 13: Sichuan; 14: Xinjiang Uygur Autonomous Region (Xinjiang); 15: Shanxi; 16: Shannxi; 17: Inner Mongolia Autonomous Region (Inner Mongolia). The species of this study from different province are shown as different shapes on the map.

The isolates of *Leishmania* sp. were collected from different foci (plain, desert and hill), and the longest distance between isolates is more than 2000 miles (from Shandong to the Xinjiang) (Figure 4). Meanwhile, different species were found in the same area. The isolate **XJ801** of *Leishmania* sp. is from Kashi city of Xinjiang. The isolate **801** identified as *L. donovani* based on ITS1 sequences by Wang *et al.*[16] and Yang *et al.*[17] is also from Kashi city. Another two isolates MHOM/CN/76/BT013 and MHOM/CN/81/812 which is differs from *L. donovani* (PHON/CN/77/771), *L. turanica* (MRHO/CN/88/KXG-2) and *L. gerbilli* (MRHO/CN/62/1) based on polymorphisms in both kinetoplast (kDNA) and nuclear (nDNA) DNAs that also collected from the same area Kashi [20]. As such, the *Leishmania* isolates in China were more heterogeneous, further epidemiologic survey and more strains are required in Kashi.

### *L. donovani* complex of China

Analysis in the current study revealed that the cyt *b* sequences of GS1, GS7 and XJ771 clustered with other species of *L. donovani* complex (PP = 1.00). On the basis of MLEE of the representative isolates from the plain, hill, and desert regions of China, Xu *et al.* were first to identify the causative agents responsible of VL as *Leishmania donovani sensu lato* and *L. infantum*[56]. The results based on sequences of cyt *b*, ITS1 [17] and COII [19] sequences clearly proved the existence of *L. donovani* in China. However, *L. donovani* or *L. infantum* standard isolates cannot be distinguished from *L. donovani* complex isolate using the cyt *b* gene in the BI and MP trees. These findings aren’t consistent the ITS1 study showing three isolates clustered with *L. donovani* and a clear classification within subspecies between *Leishmania donovani donovani* and *Leishmania donovani infantum*. Therefore, the inter-specific variation of the ribosomal RNA gene ITS1 was inferred to be more suitable than mtDNA segment cyt *b* for studying the phylogenetic relationships among subspecies. Of course, we can’t exclude the possibility that the different inter-specific variation between ITS1 and cyt *b* are calculated by choosing the different samples or numbers of the isolates or strains.

### *L. turanica* of China

Our cyt *b* data demonstrate that the isolates KXG-2 and GS-GER20 clustered with *L. turanica* (AB434675) from central Asia, findings that are congruent with those of our earlier studies [17,19] and then clustered with *L. arabica* from western Asia, a finding that agrees with that of Asato *et al.*[34] (Figures 1, 2, 3). The definitive hosts of *L. gerbilli*, *L. turanica*, *L. arabica* are rodents of the Old World [57]. Using MLEE methods, the isolate KXG-2 was identified as *L. turanica*[16], and the isolate GS-GER20 was identified as *L. gerbilli*[58]. In the 1990s, *L. turanica* and *L. gerbilli* were identified in rodents or sandflies in Karamay, Xinjiang and *L. turanica* was proved to be pathogenic in both monkeys and humans in the laboratory, *Phlebotomus mongolensis* and *Phlebotomus andrejevi* were its major vectors [16]. We considered the isolates KXG-2 and GS-GER20 to be *L. turanica* and *L. gerbilli*, respectively via the cyt *b* gene sequences.

### *L. tropica* of China

The species of the *L. tropica* complex cause the urban form of Old World CL. In Iran, Iraq, and India, it is transmitted by *Phlebotomus papatasi.* This species is rarely reported in China. The fact that the isolate JS1 was collected from Jiangsu Province clustered with *L. tropica*, which agrees with the results of our earlier study based on the COII gene [19]. Lu *et al.* used random amplified polymorphic DNA data to suggest a close relationship between the isolate JS1 and *L. tropica* (K27) [59]. Thus, we infer that the isolate JS1 may be *L. tropica*. However to further confirm this inference, more data such as host specificity, life cycle, and biochemical analysis will be needed.

### Evolution inference and epidemiology of China

In our analysis, *Leishmania* cyt *b* sequences are consistent with the genus *Leishmania* that contains three subgenera: *Leishmania*, *Sauroleishmania* and *Viannia*[4]. Based on the suggestion that mammalian *Leishmania* did not evolve from those of lizards but vice versa[60,61], Lukeš *et al.*[54] proposed that the ancestor of the new world *Leishmania* evolved in South America and then migrated via the Bering land bridge to Asia via multiple independent genetic loci. The *Leishmania* lineage would have been dispersed throughout central and/or Southeast Asia, where a major diversification gave rise to *L. aethiopica*, *L. major*, *L. gerbilli, L. turanica*, *L. tropica*, and the *L. donovani* complex. The isolates from China were absent in this analysis. However Fraga *et al.* thought this theory puts *L*. *tarentolae* (*Sauroleishmania*) in an illogical position. Our data suggest that *Leishmania* sp. of the pathogen of VL and CanL clustering with *L. tarentolae* (*Sauroleishmania*) was in the same “illogical position”. The maximum parsimony consensus tree (Figure 1) and splitstree (Figure 3) supports the idea of a common origin with the *Viannias* subgenus, whereas the Bayesian tree (Figure 2) show the Chinese/taretolae group clustered together with species of *Leishmania* subgenus. This ambiguous position of *L. tarentolae* had been discussed by Luyo-Acero *et al.* based on the same DNA marker cyt *b*[35] that *L. tarentolae* clustered with *Viannia* in the NJ tree consisting with the minicircle phylogenetic analysis [62], and clustered with *Leishmania* in the MP tree supported by ATPase 6 gene [63]. The position of *L. equatorensis* as falling outside the *Leishmania* clade in the parsimony tree is supported by the phylogeny suggested by Cupolillo *et al.*[64]. However the Chinese/tarentolae group which was not described by Lukeš *et al.*[54], may have evolved from a common ancestral parasite that came from the Americas and may split off earlier than the other OWL.

Leishmaniasis remains endemic in China, especially in the west and northwest frontier regions. The epidemic foci of VL in China were classified into three types according to different geographical origin, infective agent, and clinical evidences, i.e., plain foci, hill foci, and desert foci [20]. Human VL and CL occur in China, most being VL along with rare CL cases [56,65-67]. VL was one of the most important parasitic diseases occurring in over 17 Chinese provinces in 1951[68]. Since the condition has come under control, currently, VL is mainly prevalent in six provinces in northwest China [69] (Figure 4). This study proved that the evolution hypothesis of Tian and Chen related to the Chinese *Leishmania* isolates from different epidemic foci was limited and lacked integrity [70]. In fact, the Chinese *Leishmania* species occurs as the multiple species *L. donovani* (*L. donovani donovani*, *L. donovani infantum*), *L. turanica*, *L. tropic*, *Leishmania* sp. and so forth, and some of these such as *L. donovani* and *L. turanica* were shared with neighbouring countries including India, Russia, and Uzbekistan.

## Conclusions

The current study investigated the Chinese *Leishmania* parasites using cyt *b* sequence data. Undescribed *Leishmania* species which are clearly causative agents of CanL and human VL do exist in China and are related to the *Sauroleishmania* subgenus, may have evolved from a common ancestral parasite that came from the Americas and split off earlier than the other OWL. Our cyt *b* results also suggest the following: the isolates GS7, GS1 and XJ771 occur as part of the *L. donovani* complex; the isolate JS1 is *L. tropica*; and the isolate KXG-2 is close to the isolate GS-GER20, which is *L. turanica* and *L. gebilli* respectively. The results of the current study indicate that the isolates from China may have had a more complex evolutionary history. In the future, we will build upon the currently described data set to gain more insight into the fascinating spectrum of Chinese *Leishmania*.

## Competing interests

The authors have no competing interests to declare.

## Authors’ contributions

JPC and BBY conceived, designed and coordinated the field study, while DLC, LL, XSH and JNX participated in the study design and drafted the manuscript. All authors read and approved the final manuscript.
